# When less is not more: the effect of transparent masks on facial attractiveness judgment

**DOI:** 10.1186/s41235-023-00477-y

**Published:** 2023-04-15

**Authors:** Yongseong Lee, Su Keun Jeong

**Affiliations:** Department of Psychology, Chungbuk National University, Chungdae-ro 1, Seowon-Gu, Cheongju, 28644 Chungbuk Korea

**Keywords:** Facial attractiveness, Face masks, Transparent masks, Face occlusion, COVID-19

## Abstract

During the COVID-19 pandemic, face masks have been widely used in daily life. Previous studies have suggested that faces wearing typical masks that occlude the lower half of the face are perceived as more attractive than face without masks. However, relatively little work has been done on how transparent masks that reveal the lower half of the face affect the judgment of facial attractiveness. To investigate the effect of transparent masks on the perceived attractiveness, in the current study, we asked participants to rate the attractiveness of faces without masks and with a typical opaque mask and a transparent mask. The results showed that faces wearing opaque masks were evaluated as more attractive than those wearing transparent masks or no masks. The benefit of opaque masks was more pronounced in faces that were initially evaluated as unattractive. Interestingly, wearing transparent masks decreased the perceived attractiveness of faces but only for the faces initially rated as attractive, possibly because of the visual distortion of the lower half of the face by transparent masks. In summary, we found that opaque and transparent masks have different effects on perceived attractiveness, depending on the attractiveness of faces. Given benefits of transparent masks in socio-emotional and cognitive processing, it would be important to further understand the effect of transparent masks on face information processing.

## Significance statement

The COVID-19 pandemic has necessitated the mandatory use of face masks. The use of transparent masks has become increasingly popular. This study aims to fill the significant gap in research on transparent masks and judgments on facial attractiveness. Given the advantage of transparent masks in various domains such as speech perception, emotion recognition, and language learning, it would be important to further understand the effect of transparent masks on face information processing.

## Introduction

Face masks have been widely used because of the COVID-19 pandemic. Researchers have investigated how face masks occluding the lower half of the face influence the social and cognitive processing of information on faces. For example, face recognition has been found to be impaired with masks (Carragher & Hancock, 2020; Freud et al., 2020; Stajduhar et al., 2022). Further, when faces are covered by masks, recognizing emotions from faces becomes more difficult (Gori et al., 2021; Marini et al., 2021; but see Ruba & Pollak, 2020), and the accuracy of estimating ages from faces decreases (Thorley et al., 2022).

Another widely studied topic on how masks influence the processing of facial information is the evaluation of facial attractiveness. Recent studies have consistently reported that wearing masks improves perceived attractiveness (Hies & Lewis, 2022; Kamatani et al., 2021; Patel et al., 2020; Pazhoohi & Kingstone, 2022). The benefit of partial occlusion of faces for attractiveness judgment was observed even before the COVID-19 pandemic. Previous studies conducted before the pandemic have found that reducing information from faces, either by covering faces with an object or directly removing parts of faces, increased the facial attractiveness (Miyazaki & Kawahara, 2016; Sadr & Krowicki, 2019).

Further, the effect of occluding a face on the attractiveness judgment was context dependent. In contrast to the studies conducted during the COVID-19 pandemic, researchers in Japan showed that faces wearing sanitary masks were perceived as less attractive than those without a mask in 2016 (Miyazaki & Kawahara, 2016). The researchers argued that sanitary masks were associated with unhealthiness and thus lower ratings for attractiveness. Supporting this interpretation, the same study found that covering faces with a neutral item such as a notebook or card increased the perceived attractiveness of faces in the same study. Furthermore, wearing sanitary facial masks increased the attractiveness of faces in Japan when wearing masks was no longer strongly associated with unhealthiness during the COVID-19 pandemic (Kamatani et al., 2021).

The use of masks is an effective way to prevent the spread of COVID-19 (Abaluck et al., 2022; Gurbaxani et al., 2022). However, typical opaque masks occlude the lower parts of the face; thus, wearing masks impairs social interaction in personal or educational settings. Transparent masks that leave the lower parts of the face visible have been introduced to overcome the drawbacks of opaque masks. Using transparent masks can improve communication (Kratzke et al., 2021) and emotion recognition (Miyazaki et al., 2022). Furthermore, attention to the oral region can help children learn a language (Lewkowicz & Hansen-Tift, 2012; Tenenbaum et al., 2015). Therefore, transparent masks can be useful for children who need to develop their communication and social skills.

Despite the potential social and cognitive advantages of transparent masks, relatively little work has been done on how they affect the visual information processing of faces. For example, despite many studies using typical opaque masks, the effect of transparent masks on the judgment of facial attractiveness remains unclear. Although mask-wearing mandates have been lifted in many countries (Stokel-Walker, 2022) the widespread use of masks for more than two years could have shifted facial information processing. Indeed, a recent study demonstrated that only a few months of experience in mask-wearing was sufficient to induce changes in the patterns of processing facial information (Barrick et al., 2021). Given their advantages in various social settings, transparent masks can be widely used in the context of future pandemics. Thus, the way transparent masks would influence facial information processing compared with typical opaque masks is worth investigating.

To this end, we investigated how transparent masks affect judgments of facial attractiveness. One might predict that transparent masks would not change judgments of face attractiveness because these masks do not occlude most of the important facial features. Contrastingly, transparent masks may bias attractiveness judgment because they still partially cover the nose region and the facial contour. The results of numerous previous studies support this prediction. Reducing information by blurring the face or removing facial parts has been shown to increase perceived attractiveness (Orghian & Hidalgo, 2020; Patel et al., 2020; Pazhoohi & Kingstone, 2022; Sadr & Krowicki, 2019). In this scenario, faces wearing transparent masks would be perceived as more attractive than faces without masks, depending on the size of the occluded facial area. A third possibility is that faces with transparent masks would be perceived as less attractive than faces without masks, because the visible region in the transparent mask may bias attractive judgment by changing the facial outline and perception of the skin (Fink et al., 2001; Hong Liu & Chen, 2018; Russell, 2003; Russell et al., 2016). To find the answer, we asked participants to evaluate the attractiveness of faces with no mask, a transparent mask, or an opaque mask. Because we thought all three predictions were plausible, Experiment 1 was exploratory. In Experiment 1, we made a transparent mask by removing the mouth region of an opaque facial mask. Experiments 2 and 3 made different types of transparent masks to replicate and generalize the result of Experiment 1. Specifically, after removing the mouth region of an opaque facial mask, we overlaid a photo of transparent plastic film on the mouth region of an opaque mask to simulate visual distortion caused by transparent masks in Experiment 2, and photos of real transparent masks were used in Experiment 3.

## Experiment 1

### Method

#### Participants

Hies and Lewis (2022) reported an $\eta_{p}^{2}$ of 0.32 when comparing four facial occlusion conditions. In the current study, we used Cohen’s *f* of 0.6859 (equivalent to an $\eta_{p}^{2}$ of 0.32), an alpha level of 0.05, a power of 0.9. A power analysis using G*Power 3 revealed that seven participants are needed when comparing three conditions (Faul et al., 2007). Nevertheless, we increased the sample size to 40 to ensure sufficient statistical power.

Forty participants were recruited from Amazon Mechanical Turk (MTurk). To participate in the experiment, the MTurk workers were required to be U.S. residents and were required to have the Masters qualifications and an approval rate of at least 98% in past studies. One participant who did not follow the instruction was excluded from further analyses. Data from the remaining 39 participants were analyzed.

### Materials

Following Hies and Lewis (2022), we selected 20 attractive and 20 unattractive male face images from the Chicago Face Database (CFD) (Ma et al., 2015). The average attractive ratings for the attractive and unattractive faces were 3.820 (SD = 0.347) and 2.194 (SD = 0.223), respectively. The mean age of the faces was 26.964 years (SD = 5.657). All the images were frontal views with neutral facial expressions.

**Fig. 1:**
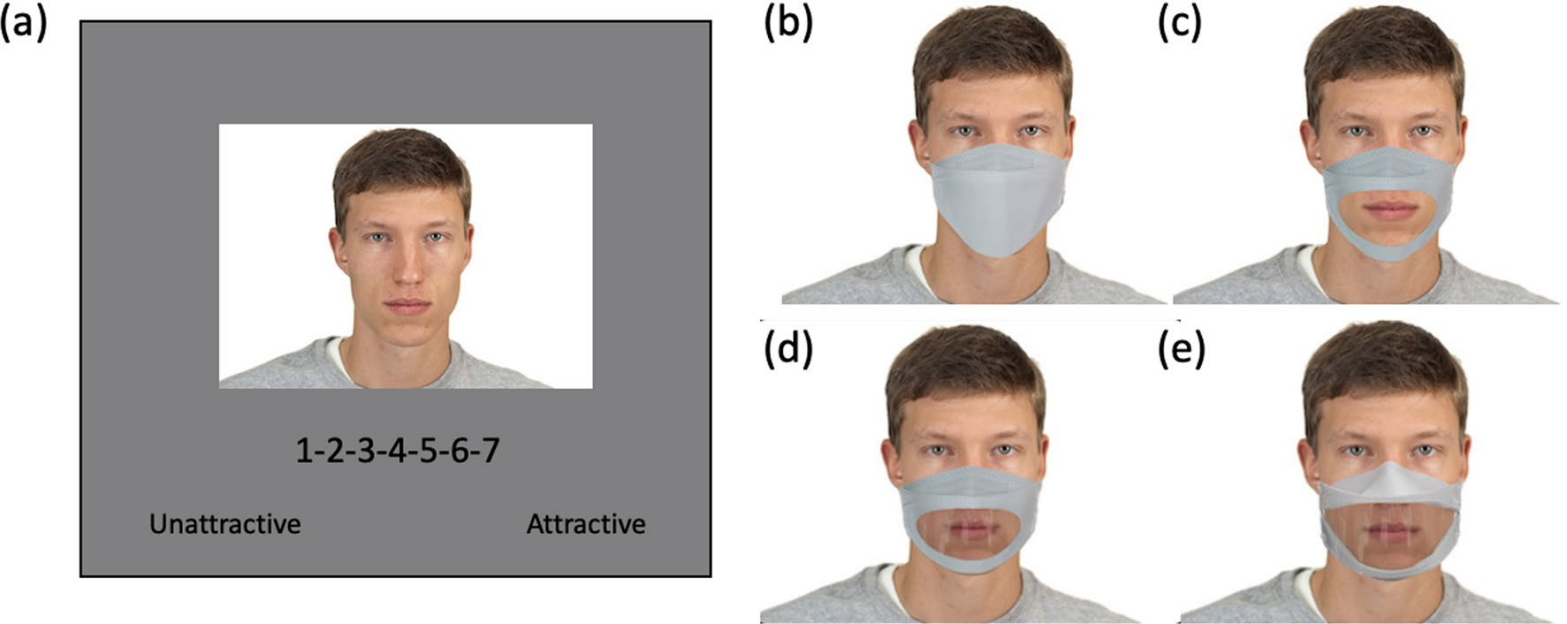
**a** Example trial of the experiment. Images are not drawn to scale. **b** and **c** Example stimuli of the opaque mask and transparent mask conditions in Experiment 1. **d** and **e** Example stimuli of the transparent mask in Experiments 2 and 3

We manually overlaid an opaque mask image on each face image using the Adobe Photoshop software (Fig. 1b). The size of the mask was adjusted to fit the shape of each face. To make transparent mask stimuli, we removed the mouth region of an opaque mask and superimposed the mask on each face image (Fig. 1c). Although the mouth region of a face can be clearly seen through a transparent mask, seeing through transparent media is not identical to seeing without any barrier. Transparent coverings can slightly reflect light and impair the quality of the visual information behind them (Erber, 1979; Singh et al., 2021). To simulate the slight distortion of the visual information through the transparent surface, we added Gaussian blur to the mouth region of the transparent mask. The size of face images varied between 1344 × 945 and 1955 × 1374 pixels. A Gaussian filter with 4 pixels radius was applied to the mouth region of each face image, and the images were scaled down to fit 30% of the screen height.

The final stimuli consisted of 40 no masks, 40 transparent masks, and 40 opaque mask faces.

#### Procedure

The experiment was programmed using the PsychoPy (Peirce et al., 2019). The participants were recruited via MTurk and redirected to the Pavlovia.org website where the experiment was hosted. The participants were asked to respond to an online informed consent form and then were shown an instruction screen. They were asked to rate the attractiveness of a given face on a scale from 1 to 7 using the number keys on the keyboard (Fig. 1a). In each trial, a face with no mask, opaque mask, or transparent mask was presented in random order.

Because the participants used their own devices, we could not precisely control the stimulus size, screen size, or viewing distances. Thus, we presented the face image to occupy 30% of the screen height. A face image was presented after a 500 ms fixation and remained on the screen until response. A text message that indicated a 1–7 rating scale appeared immediately below the face image. The font size was 3.5% of the screen’s height.

### Results

A repeated-measures ANOVA with base attractiveness (attractive and unattractive) and mask type (no mask, transparent mask, and opaque mask) as factors was performed on the perceived attractiveness ratings. A Greenhouse–Geisser correction was applied when the sphericity assumption was violated. All post-hoc tests were Bonferroni-corrected.

**Fig. 2:**
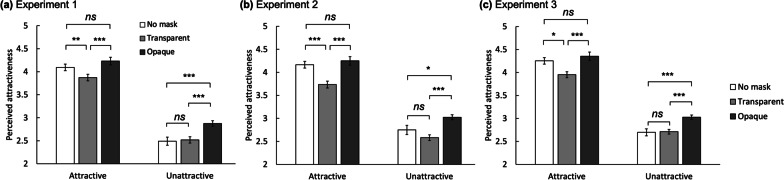
Average perceived attractiveness for the conditions without a mask, with a transparent mask, and with an opaque mask grouped by the base attractiveness ratings. The error bars represent within-subject standard errors. *p < .05, **p < .01, ***p < .001, *ns* Not significant

The result showed a significant main effect of base facial attractiveness, *F*(1, 38) = 123.682, *p* < 0.001, $\eta_{p}^{2}$ = 0.765. The main effect of mask type was also significant, *F*(1.850, 70.291) = 21.599, *p* < 0.001, $\eta_{p}^{2}$ = 0.362. Post-hoc analysis revealed that faces with an opaque mask were evaluated as more attractive than faces without a mask, *t*(38) = 4.641, *p* < 0.001, *d* = 0.282. Furthermore, faces with an opaque mask were rated higher than those with a transparent mask, *t*(38) = 6.351, *p* < 0.001, *d* = 0.386. However, wearing transparent masks and not wearing masks did not show a significant difference, *t*(38) = 0.170, *p* = 0.274, *d* = 0.104 (Fig. 2a). Interestingly, there was a significant interaction between base attractiveness and mask type, *F*(1.732, 65.812) = 9.957, *p* < 0.001, $\eta_{p}^{2}$ = 0.208.

To scrutinize the interaction effect, we analyzed attractive and unattractive faces separately. For attractive faces, a significant main effect of mask type was found, *F*(1.873, 71.165) = 13.883, *p* < 0.001, $\eta_{p}^{2}$ = 0.268. In a post-hoc test, no significant difference was found between the attractive ratings of faces with no mask and those with an opaque mask, *t*(38) = − 2.011, *p* = 0.144, *d* = − 0.133. This result is consistent with that of previous studies that reported little or no advantage of facial masks when a face was highly attractive (Patel et al., 2020). In contrast, faces with transparent masks were rated as less attractive than both those with no mask (*t*(38) = − 3.212, *p* = 0.006, *d* = − 0.212) and faces with opaque masks (*t*(38) = − 5.233, *p* < 0.001,* d* = − 0.345), showing that transparent masks decreased facial attractiveness.

For unattractive faces, the effect of mask type was also significant, *F*(1.436, 54.585) = 25.030, *p* < 0.001, $\eta_{p}^{2}$ = 0.397. Post-hoc tests showed that the opaque mask condition had higher attractiveness ratings than both the no mask condition (*t*(38) = 6.347, *p* < 0.001, *d* = 0.483) and transparent mask condition (*t*(38) = 5.881, *p* < 0.001, *d* = 0.447). The no mask and transparent mask conditions, however, did not differ significantly, *t*(38) = − 0.467, *p* > 0.99, *d* = − 0.035.

Response time (RT) data were analyzed in the same manner. The main effect of base attractiveness was marginally significant, *F*(1, 38) = 2.901, *p* = 0.097, $\eta_{p}^{2}$ = 0.071. Other main effect and interaction were not significant (the main effect of mask type: *F*(1.231, 46.782) = 1.953, *p* = 0.167, $\eta_{p}^{2}$ = 0.049; mask type × base attractiveness interaction: *F*(1.190, 45.203) = 1.655, *p* = 0.206, $\eta_{p}^{2}$ = 0.042).

When RT data were analyzed separately for attractive and unattractive face groups, no significant difference was found across the no mask, transparent mask, and opaque mask conditions for both face groups (attractive group, *F*(1.099, 41.759) = 1.948, *p* = 0.170, $\eta_{p}^{2}$ = 0.049; unattractive group, *F*(2, 76) = 0.849, *p* = 0.432, $\eta_{p}^{2}$ = 0.022).

## Experiment 2

We found that transparent masks reduced facial attractiveness for attractive faces in Experiment 1. In Experiment 1, we made transparent masks by removing the mouth region of opaque masks. We applied a Gaussian blur filter to the mouth region of transparent masks. Nevertheless, this manipulation may not accurately simulate visual distortion such as light reflection caused by real transparent masks. Therefore, in Experiment 2, to increase the ecological validity of transparent mask images, we removed the mouth region of an opaque mask image and superimposed a photo of clear plastic film taken from photos of real transparent masks on each face image.

### Method

#### Participants

Forty participants were recruited from Amazon Mechanical Turk (MTurk), using the same criteria in Experiment 1.

### Materials

To make transparent mask images, we first overlaid a photo of an opaque mask on each face image and removed the mouth region as was done in Experiment 1. Then, we took a photo of a real transparent mask and cropped the clear plastic film part of the mask. The background behind the clear film was manually removed by adjusting image properties using Adobe Photoshop software. Specifically, we manually selected the clear film to create a layer mask and then adjusted the density (i.e., opacity) of the layer mask to 63% with a feathering of 33 pixels to remove the background. The remaining clear plastic texture was superimposed on the mouth region of the opaque mask (Fig. 1d). Thus, the shape of the mask was the same between the opaque and transparent mask conditions. Stimuli in opaque mask and no mask conditions were the same as those in Experiment 1.

#### Procedure

The experimental procedure was the same as that in Experiment 1.

### Results

We analyzed the data using the same method in Experiment 1. A repeated-measures ANOVA with base attractiveness (attractive and unattractive) and mask type (no mask, transparent mask, and opaque mask) as factors on the perceived attractive rating revealed significant main effects of bas attractiveness and mask type, *F*(1, 39) = 184.106, *p* < 0.001, $\eta_{p}^{2}$ = 0.825 and *F*(1.637, 63.825) = 10.797, *p* < 0.001, $\eta_{p}^{2}$ = 0.217, respectively. Interaction between attractiveness and mask type was significant, replicating the result of Experiment 1, *F*(2, 78) = 12.422, *p* < 0.001, $\eta_{p}^{2}$ = 0.242.

For attractive faces, a main effect of mask type was found, *F*(1.730, 67.463) = 11.895, *p* < 0.001, $\eta_{p}^{2}$ = 0.234. In post-hoc analyses, the opaque and no mask conditions did not significantly differ, *t*(39) = − 0.739, *p* > 0.99, *d* = − 0.083. However, the transparent mask condition showed lower attractive ratings than both the no mask and opaque mask conditions, *t*(39) = 3.806, *p* < 0.001, *d* = 0.429 and *t*(39) = − 4.545, *p* < 0.001, *d* = − 0.512, respectively.

A significant effect of mask type was also found in unattractive faces, *F*(1.608, 62.720) = 9.667, *p* < 0.001, $\eta_{p}^{2}$ = 0.199. Faces with opaque masks were rated as more attractive than those with no mask and transparent masks, *t*(39) = 2.706, *p* = 0.025, *d* = 0.323 and t(39) = 4.354, *p* < 0.001, *d* = 0.520, respectively. In contrast, no mask and transparent mask conditions did not show a significant difference, *t*(39) = 1.648, *p* = 0.310, *d* = 0.197.

RT data showed no significant effect (the main effect attractiveness: *F*(1, 39) = 0.767, *p* = 0.387, $\eta_{p}^{2}$ = 0.019; the main effect of mask type:* F*(1.218, 47.520) = 1.008, *p* = 0.337, $\eta_{p}^{2}$ = 0.025; mask type $\times$ base attractiveness interaction: F(2, 78) = 0.487, *p* = 0.616, $\eta_{p}^{2}$ = 0.012). When RT data were analyzed for attractive and unattractive faces, no significant difference was found across the no mask, transparent mask, and opaque mask conditions (attractive group, *F*(1.570, 61.220) = 0.826, *p* = 0.417, $\eta_{p}^{2}$ = 0.021; unattractive group, *F*(1.146, 44.683) = 0.896, *p* = 0.363, $\eta_{p}^{2}$ = 0.022).

## Experiment 3

In Experiment 2, we created transparent mask images by removing the mouth region of an opaque mask and superimposing a photo of transparent plastic film. We tried to match the shape of the mask between opaque and transparent mask conditions in Experiment 2 by using the same opaque mask images. However, the transparent masks in Experiment 2 still may look artificial, because we subjectively defined and removed the mouth region of an opaque mask. Therefore, we conducted Experiment 3 to further test the validity of the mask stimuli and replicate Experiments 1 and 2. In Experiment 3, we overlaid photos of a real transparent mask on the face images, instead of editing the mouth region of an opaque mask.

### Method

#### Participants

Forty participants were recruited from Amazon Mechanical Turk (MTurk), using the same criteria in Experiment 1. One participant who did not follow the instruction was excluded and data from the remaining 39 participants were analyzed.

### Materials

To replicate and generalize Experiments 1 and 2, we used a new set of transparent mask images. In Experiments 1 and 2, we manipulated the mouth region of an opaque mask. However, in Experiment 3, we took a photo of a real transparent mask and manually removed the background behind the transparent mouth region. Then, the transparent mask image itself was superimposed on each face to create transparent mask stimuli (Fig. 1e). As a result, the shape of masks was slightly different between the opaque and transparent mask conditions in Experiment 3 (see Fig. 1d and e). However, we try to increase the ecological validity of stimuli by using real transparent mask photos.”

#### Procedure

The experimental procedure was the same as those in Experiments 1 and 2.

### Results

Replicating the results of Experiments 1 and 2, we found significant main effects of base attractiveness and mask type, *F*(1, 38) = 252.110, *p* < 0.001, $\eta_{p}^{2}$ = 0.869 and *F*(1.365, 51.870) = 9.566, *p* < 0.001, $\eta_{p}^{2}$ = 0.201, and a significant interaction between the two factors, *F*(1.601, 60.820) = 9.042, *p* < 0.001, $\eta_{p}^{2}$ = 0.192.

For attractive faces, the effect of mask type was significant, *F*(1.497, 56.896) = 7.519, *p* = 0.003, $\eta_{p}^{2}$ = 0.165. The opaque mask and no mask conditions did not differ, *t*(38) = − 0.938, *p* > 0.99, d = − 0.109. However, the transparent mask condition showed lower attractiveness ratings compared to the no mask and opaque mask conditions, *t*(38) = 2.790, *p* = 0.020, *d* = 0.325 and *t*(38) = − 3.728, *p* < 0.001, *d* = − 0.434, respectively.

For unattractive faces, a significant main effect of mask type was found, *F*(1.369, 52.038) = 14.108, *p* < 0.001, $\eta_{p}^{2}$ = 0.271. The no mask and transparent mask conditions did not show a significant difference, *t*(38) = − 0.201, *p* > 0.99, *d* = − 0.015, but faces with opaque masks were rated as more attractive than those with no mask and transparent masks, *t*(38) = 4.697, *p* < 0.001, *d* = 0.347 and *t*(38) = 4.496, *p* < 0.001, *d* = 0.332, respectively.

RT for attractive faces was slower than that for unattractive faces, *F*(1, 38) = 4.533, *p* = 0.040, $\eta_{p}^{2}$ = 0.107 (slower attractive), but the main effect of mask type and interaction between attractiveness and mask type were not significant, *F*(1.351, 51.321) = 0.688, *p* = 0.452, $\eta_{p}^{2}$ = 0.018 and *F*(1.288, 48.946) = 2.789, *p* = 0.092, $\eta_{p}^{2}$ = 0.068, respectively. No main effect of mask type was found when RT data were analyzed separately for attractive and unattractive faces (attractive group, *F*(1.185, 45.039) = 1.955, *p* = 0.167, $\eta_{p}^{2}$ = 0.049; unattractive group, *F*(1.593, 60.546) = 1.061, *p* = 0.339, $\eta_{p}^{2}$ = 0.027).

## General discussion

In this study, we investigated how transparent masks that do not occlude the mouth region and opaque masks that cover the entire lower half of a face affect the judgments of facial attractiveness. The results showed that opaque masks increased the perceived attractiveness of faces. This benefit was more pronounced for the unattractive faces. Furthermore, we found that wearing transparent masks decreased the perceived attractiveness of faces. Interestingly, the effect of transparent masks was observed only for the attractive faces. These results were replicated across three experiments that used different types of transparent masks. In summary, unattractive faces were perceived as more attractive when wearing opaque masks and attractive faces were perceived as less attractive when wearing transparent masks.

The current study replicated previous studies that reported the effect of opaque masks on the perceived attractiveness of faces (Hies & Lewis, 2022; Kamatani et al., 2021; Orghian & Hidalgo, 2020; Patel et al., 2020; Pazhoohi & Kingstone, 2022). There are several potential explanations for why faces wearing masks are rated higher. For example, faces occluded by masks look better because masks hide facial defects or increase facial symmetry by covering asymmetrical features (Patel et al., 2020). Further, humans tend to complete the part covered by masks with average faces which are sometimes more attractive than the original part (Kramer & Jones, 2022).

However, in the present study, only faces that were originally evaluated as unattractive were found to benefit from opaque masks. The ratings for the attractive faces increased numerically when opaque masks were applied, but the improvement was not statistically significant after correcting for multiple comparisons. This result does not contradict those of previous studies (Miyazaki & Kawahara, 2016; Patel et al., 2020; Pazhoohi & Kingstone, 2022). For instance, in Patel et al. (2020), attractiveness ratings for individuals who were initially rated as unattractive without wearing a mask improved by almost 40% after wearing an opaque mask. In contrast, those who were initially rated attractive without a mask showed less than 10% improvements after using the opaque face mask. Some attractive individuals even received lower ratings of attractiveness when an opaque face mask covered the lower half of the face. These results can be explained by the regression toward the mean.

Our main objective was to investigate the effect of transparent masks on perceived attractiveness. One would predict that transparent masks would increase the perceived attractiveness of faces because reducing facial information tends to increase attractiveness (Kramer & Jones, 2022; Orghian & Hidalgo, 2020; Patel et al., 2020; Sadr & Krowicki, 2019). However, we found that faces that were initially rated as attractive were perceived as less attractive when transparent masks were applied.

Unlike other types of visual information, face processing is known to rely on holistic processing (Young et al., 1987) and, thus, inverting a face disrupts the performance of face recognition (Yin, 1969). However, recent studies have found that the face inversion effect was reduced in faces wearing opaque masks, suggesting that masks disrupted holistic processing (Freud et al., 2020; Stajduhar et al., 2022). Previous studies have argued that holistic processing plays an important role in the facial attractiveness evaluation (Abbas & Duchaine, 2008; Hong Liu & Chen, 2018). Accordingly, one might argue that although transparent masks do not cover the entire lower half of the face, they still disrupt the holistic processing of faces, which could have contributed to the lower perceived attractiveness. However, both wearing transparent masks and not wearing masks showed the same amount of face inversion effect (Lee, 2022), suggesting that transparent masks do not significantly impair holistic processing.

Instead, transparent masks can reduce the ratings of attractiveness of individuals because they distort facial features in the evaluations of attractiveness. Facial symmetry has been found to influence attractiveness (Grammer & Thornhill, 1994; Perrett et al., 1999). The mouth seen through the transparent surface of the mask could be slightly misaligned with the center, resulting in the increased asymmetry of facial features. Further, the outline of the lower half of the face wearing transparent masks may appear shrunken. Therefore, the altered outline of the lower half of the face decreased the perceived attractiveness (Hong Liu & Chen, 2018).

Other visual attributes of a face could influence the evaluation of attractiveness. We added a Gaussian blur filter to the mouth region of the transparent mask, superimposed transparent plastic film on the mouth region, and used photos of real transparent masks to model a slight distortion of light induced by the transparent surface of the mask. These manipulations made the skin around the mouth region look different from those around other parts of the face. As skin color, texture, and reflections can influence facial attractiveness (Fink et al., 2001; Russell, 2003), visual distortion by transparent masks could have made faces perceived as less attractive.

Before the COVID-19 pandemic, wearing sanitary masks was associated with the disease in Japan and did not improve perceived attractiveness (Miyazaki & Kawahara, 2016). However, sanitary masks improved attractiveness after the COVID-19 pandemic as masks no longer indicated unhealthiness (Kamatani et al., 2021). Similarly, medical masks enhanced facial attractiveness even more than other types of masks (Hies & Lewis, 2022), possibly because of the positive association between medical masks and medical professionals during the pandemic. Therefore, transparent masks, which are not as common as typical opaque masks, can be perceived as not properly covering the mouth region. This interpretation may lead to a negative impression of faces when wearing transparent masks.

Interestingly, the perceived attractiveness decreased in faces that wore transparent masks—only in faces that were initially rated attractive. Transparent masks neither improved nor impaired the perceived attractiveness of faces initially rated as unattractive. We speculate that this was due to the floor effect or response bias. The rating for the no mask condition was already low with not enough room to go further below. Also, the participants could have avoided extreme responses (i.e., responding 1), showing a similar pattern to the regression toward the mean.

Further studies are needed to confirm the factors that contributed to the perceived attractiveness of faces with transparent masks. Our study used transparent masks that covered the nose region and facial outline. In contrast, studies that investigated the effect of transparent masks on emotion recognition used a mask that partly covered the nose and revealed most of the lower half of the face (McCrackin et al., 2022; Miyazaki et al., 2022). It would be interesting to examine how the effect of transparent masks changes depending on the size of the facial area occluded by them.

In summary, our study demonstrated that typical opaque masks and transparent masks have different effects on perceived facial attractiveness. While opaque masks increased attractiveness for unattractive faces, transparent masks decreased attractiveness only for attractive faces. Many countries have lifted mask mandates (Stokel-Walker, 2022). Nevertheless, owing to the effectiveness of masks in preventing the spread of COVID-19 (Leffler et al., 2020), mask-wearing may be a reasonable response to a potential pandemic. Given the advantage of transparent masks in various domains such as speech perception, emotion recognition, and language learning, it would be important to further understand the effect of transparent masks on face information processing.

## Data Availability

The datasets used and/or analyses are available from the corresponding author on reasonable request.

## Abbreviations

MTurk: Amazon mechanical turk
CFD: Chicago face database
RT: Response time
